# Supplementary material for: Cardiac-specific loss of mitoNEET expression is linked with age-related heart failure
Source: Commun Biol. 2021 Jan 29;4:138. doi: 10.1038/s42003-021-01675-4 (PMC7846856; doi:10.1038/s42003-021-01675-4)

Cardiac-specific loss of mitoNEET expression is linked with age-related heart failure

Takaaki Furihata, Shingo Takada, Naoya Kakutani, Satoshi Maekawa, Masaya Tsuda, Junichi Matsumoto, Wataru Mizushima, Arata Fukushima, Takashi Yokota, Nobuyuki Enzan, Shouji Matsushima, Haruka Handa, Yoshizuki Fumoto, Junko Nio-Kobayashi, Toshihiko Iwanaga, Shinya Tanaka, Hiroyuki Tsutsui, Hisataka Sabe, and Shintaro Kinugawa

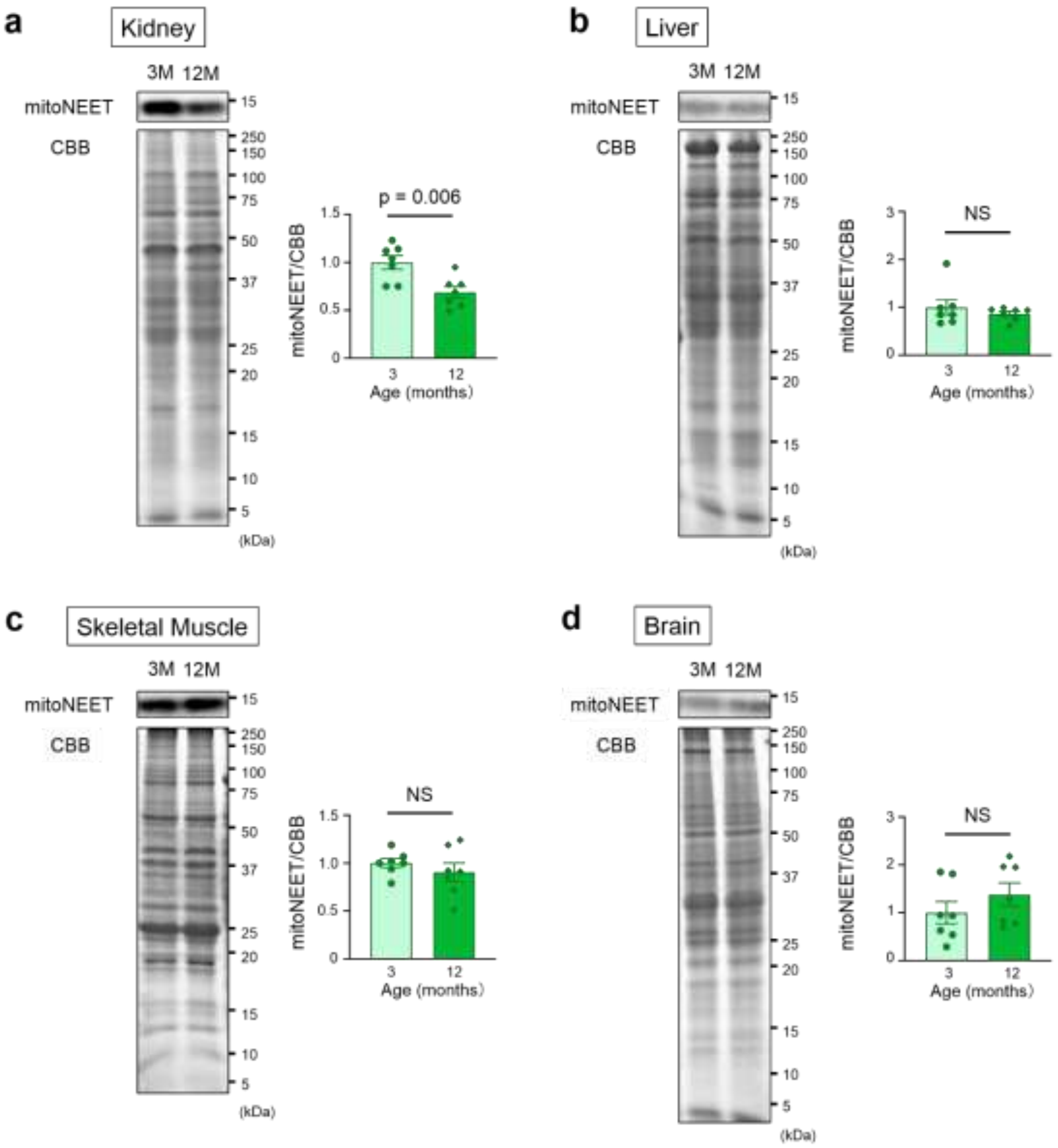

Supplementary Figure 1. MitoNEET protein expression in various organs

Representative immunoblot and summary data of mitoNEET protein expression in the kidney (**a**), liver (**b**), skeletal muscle (**c**), and brain (**d**) of C57BL/6J mice at 3 months (n = 7) and 12 months (n = 7) of age. Data are shown as the mean ± s.e; individual data points are shown. The Student unpaired two-tailed *t*-test was performed to compare means between 2 groups. ; CBB, Coomassie Brilliant Blue; NS, not significant.

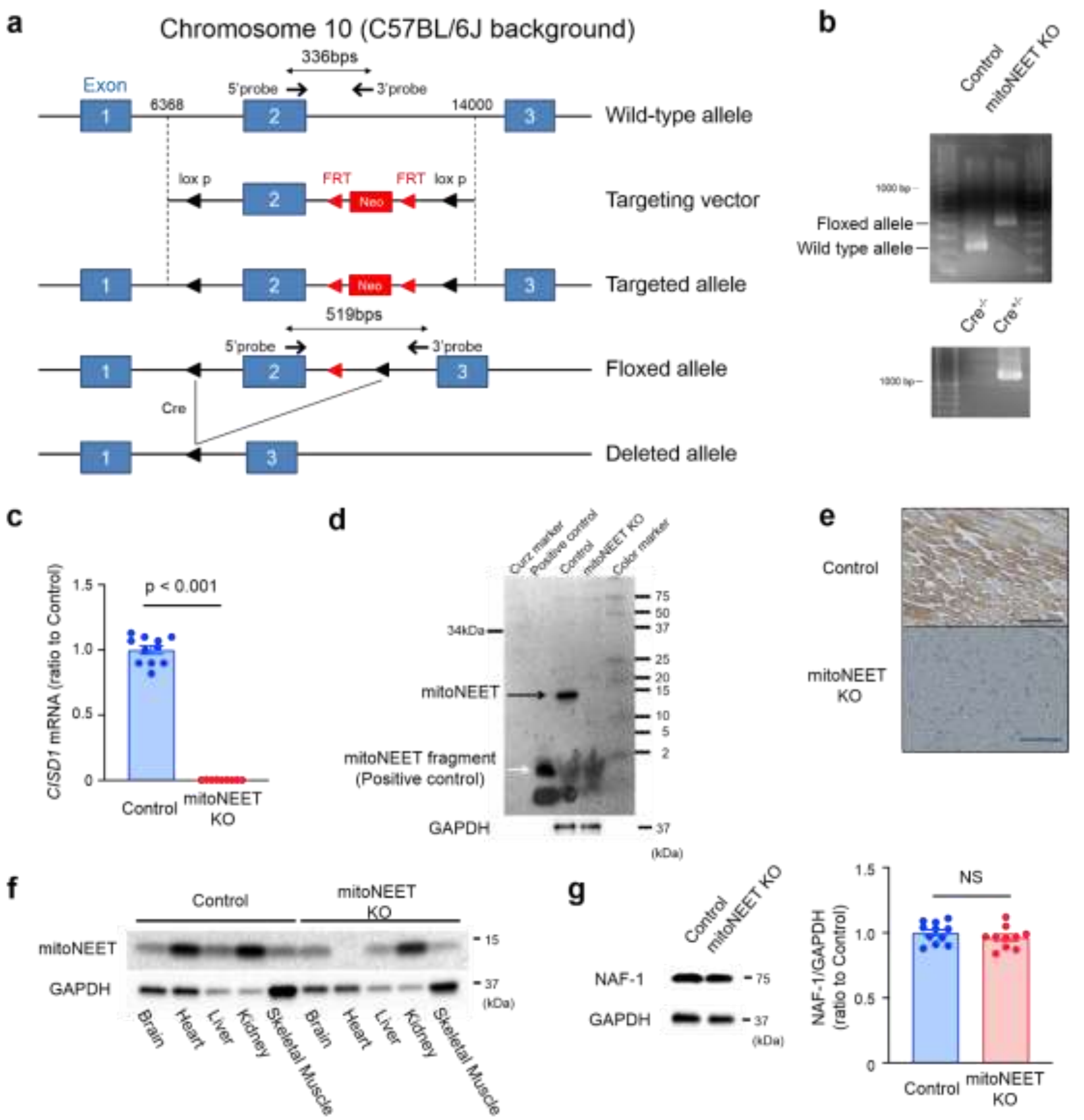

Supplementary Figure 2. Method of generation of cardiac-specific mitoNEET KO mice

(a) Design of the mitoNEET targeting construct and the genomic structure of mitoNEET. LoxP sites were inserted to delete the entire exon 2, resulting in early termination and truncation of the C-terminal region of mitoNEET. This resulted in the complete destruction of mitoNEET function. The indicated primers were used for detecting the mitoNEET flox allele. (**b)** Wild-type and mitoNEET floxed alleles were distinguished by PCR analysis. Genomic PCR confirmed the mitoNEET floxed allele

and Cre allele in mitoNEET KO mice. **(c)** Quantitative analysis of the expression of *CISD1* mRNA in the hearts of Control (n = 11) and mitoNEET KO mice (n = 10). Data are shown as the mean ± s.e.; individual data points are shown. *$p < 0.05$ vs. Control (two-tailed *t*-test). (**d)** Representative immunoblots of Tris-Tricine SDS-PAGE lysates from Control and mitoNEET KO mice, and the mitoNEET peptide fragment. The black arrow (about 14 kDa) indicates mitoNEET, and the white arrow (just below 2 kDa) indicates the mitoNEET fragment used as a positive control. **(e)** Representative immunostaining for mitoNEET in myocardial sections. Scale bar, 50 µm. (**f**) Representative immunoblot of the mitoNEET protein in various organs from mitoNEET KO mice and Control mice. (g) Representative immunoblot and summary data of Miner1 protein expression normalized to GAPDH in the hearts of Control (n = 11) and mitoNEET KO mice (n = 10). Data are shown as the mean ± s.e.; individual data points are shown. The Student unpaired two-tailed *t*-test was performed to compare means between Control and mitoNEET KO mice. GAPDH, glyceraldehyde phosphate dehydrogenase. NS, not significant.

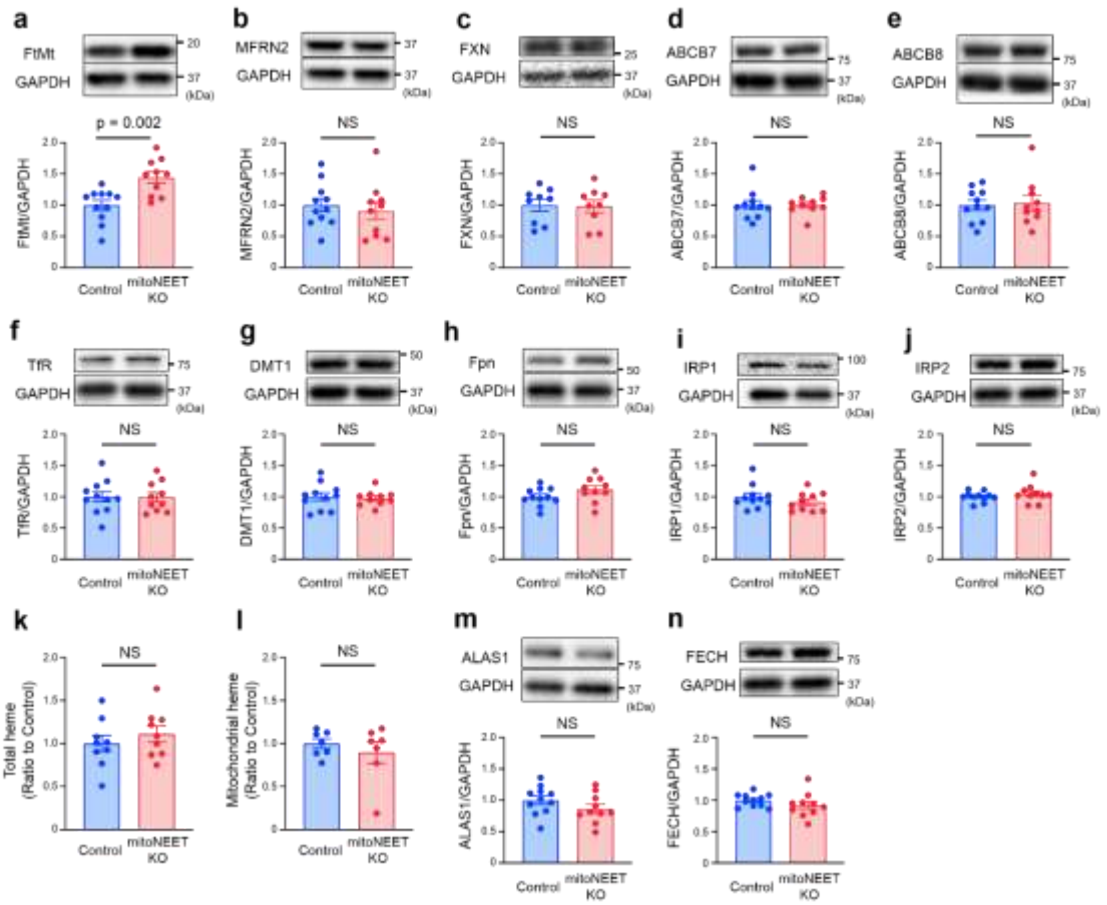

Supplementary Figure 3. Expression levels of proteins associated with iron homeostasis and heme synthesis in the heart

Representative immunoblots and summary data of the protein expression of FtMt (**a**), MFRN2 (**b**), FXN (**c**), ABCB7 (**d**), ABCB8 (**e**), TfR (**f**), DMT1 (**g**), Fpn (**h**), IRP1 (**i**), and IRP2 (**j**) normalized to GAPDH in the hearts of Control (n = 11) and mitoNEET KO mice (n = 10). Levels of total heme (**k**) and mitochondrial heme (**l**) in mitoNEET KO mice (n = 9) relative to Control mice (n = 9). Representative immunoblots and summary data of the protein expression of ALAS1 (**m**) and FECH (**n**) normalized to GAPDH in the hearts of Control (n = 11) and mitoNEET KO mice (n = 10). Data are shown as the mean ± s.e.; individual data points are shown. The Student unpaired two-tailed *t*-test was performed to compare means between Control and mitoNEET KO mice

of identical age. GAPDH, glyceraldehyde phosphate dehydrogenase; FtMt, mitochondrial ferritin; MFRN2, mitoferrin-2; FXN, frataxin; ABCB7, ATP-binding cassette protein B7; ABCB8, ATP-binding cassette protein B8; TfR, transferrin receptor; DMT1, divalent metal transporter 1; Fpn, ferroportin; IRP1, iron regulatory protein 1; IRP2, iron regulatory protein 2; ALAS1, 5'-aminolevulinate synthase 1; FECH, ferrochelatase; NS, not significant

Supplementary Table 1. Characteristics of the mice used in this study

Age	3 months		12 months		16 months	
	Control	mitoNEET KO	Control	mitoNEET KO	Control	mitoNEET KO
n	11	10	8	9	5	5
BW (g)	19.4 ± 0.3	19.6 ± 0.5	28.6 ± 1.6	28.8 ± 1.8	33.8 ± 2.5	32.5 ± 0.9
LVW/BW (mg/g)	3.5 ± 0.2	3.2 ± 0.1	2.8 ± 0.1	3.7 ± 0.4*	2.6 ± 0.1	3.5 ± 0.2*
Lung weight/BW (mg/g)	7.2 ± 0.1	6.7 ± 0.2	6.3 ± 0.3	6.9 ± 0.7	4.6 ± 0.2	5.8 ± 0.4*

Data are shown as the mean ± s.e.; *$p < 0.05$ vs. Controls of the same age (two-tailed *t*-test). KO, knockout; BW, body weight; LVW, left ventricular weight

Original Western blots in Figure 1a and Figure 5c and d

Figure 1

a

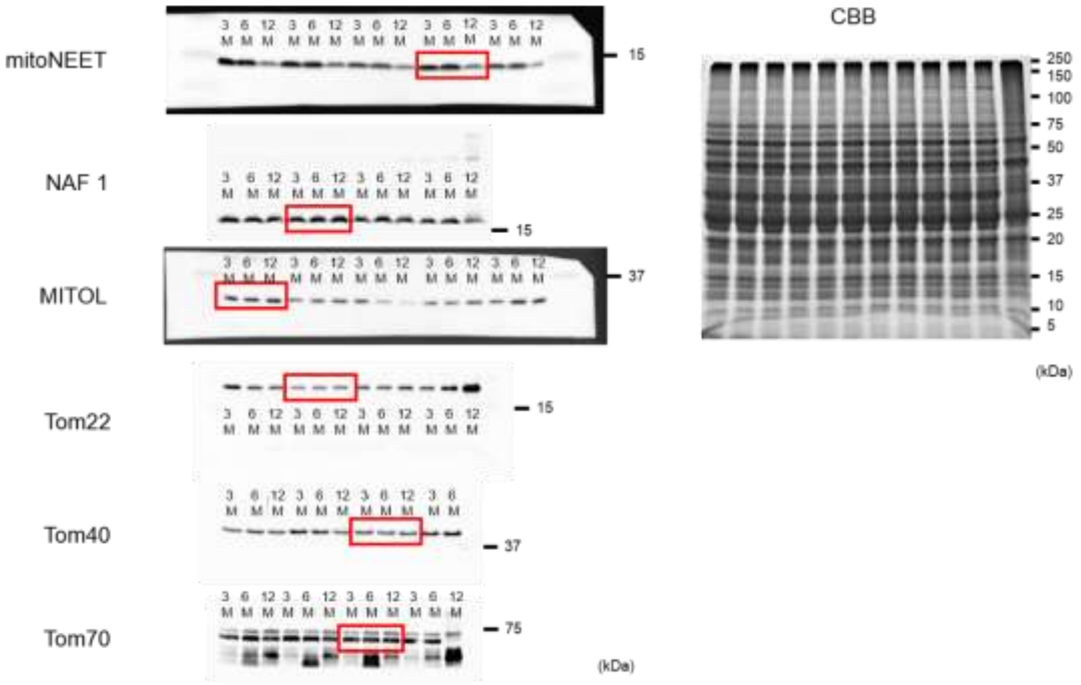

Figure 5

c, d

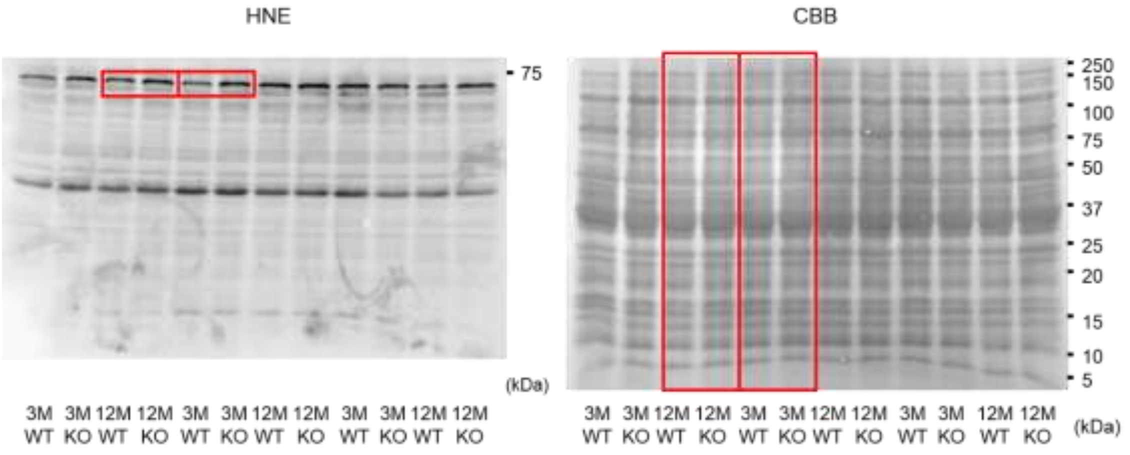

Original Western blots in Supplementary Figure 1a-d and 2b

Supplemental Figure 1

a - d

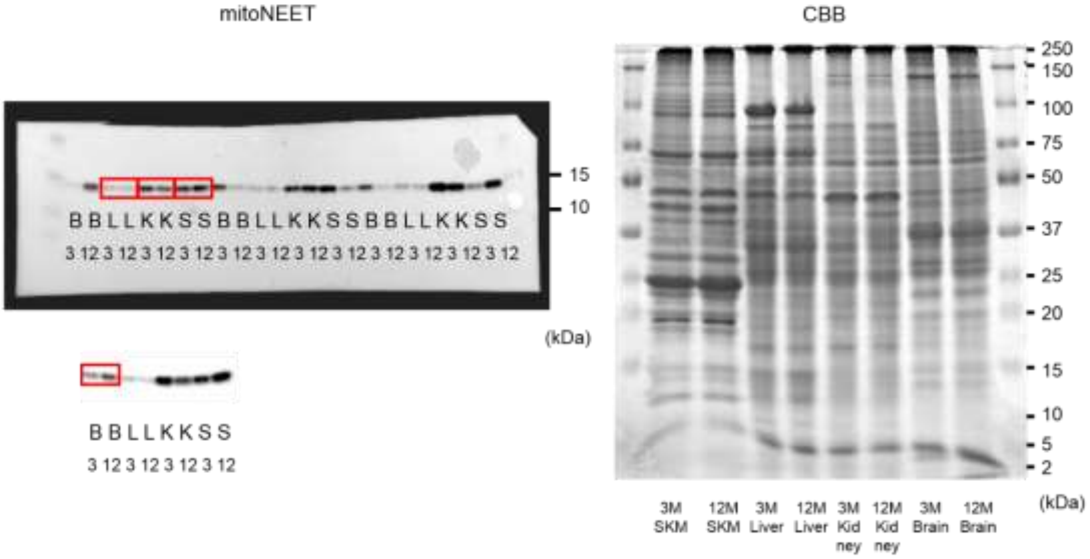

Supplemental Figure 2

b

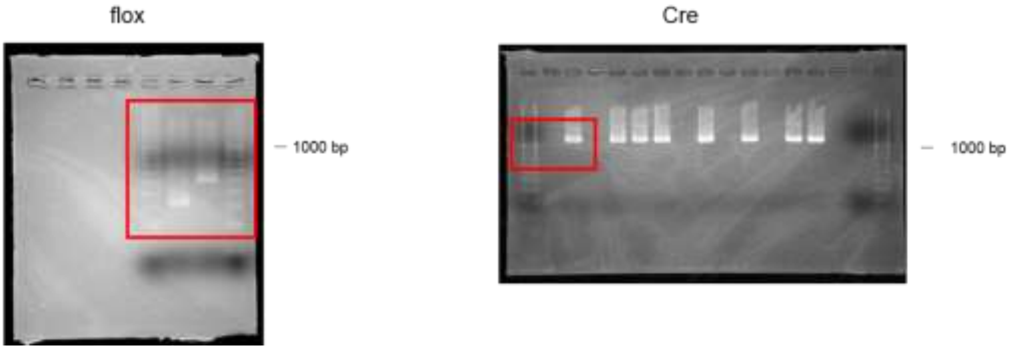

Original Western blots in Supplementary Figure 2d, f, and g

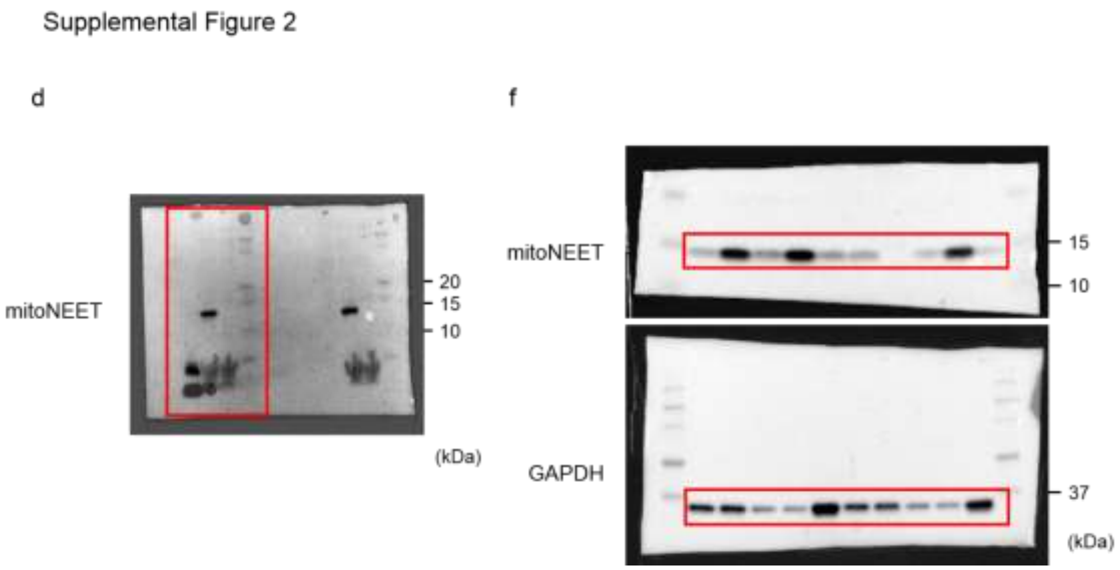

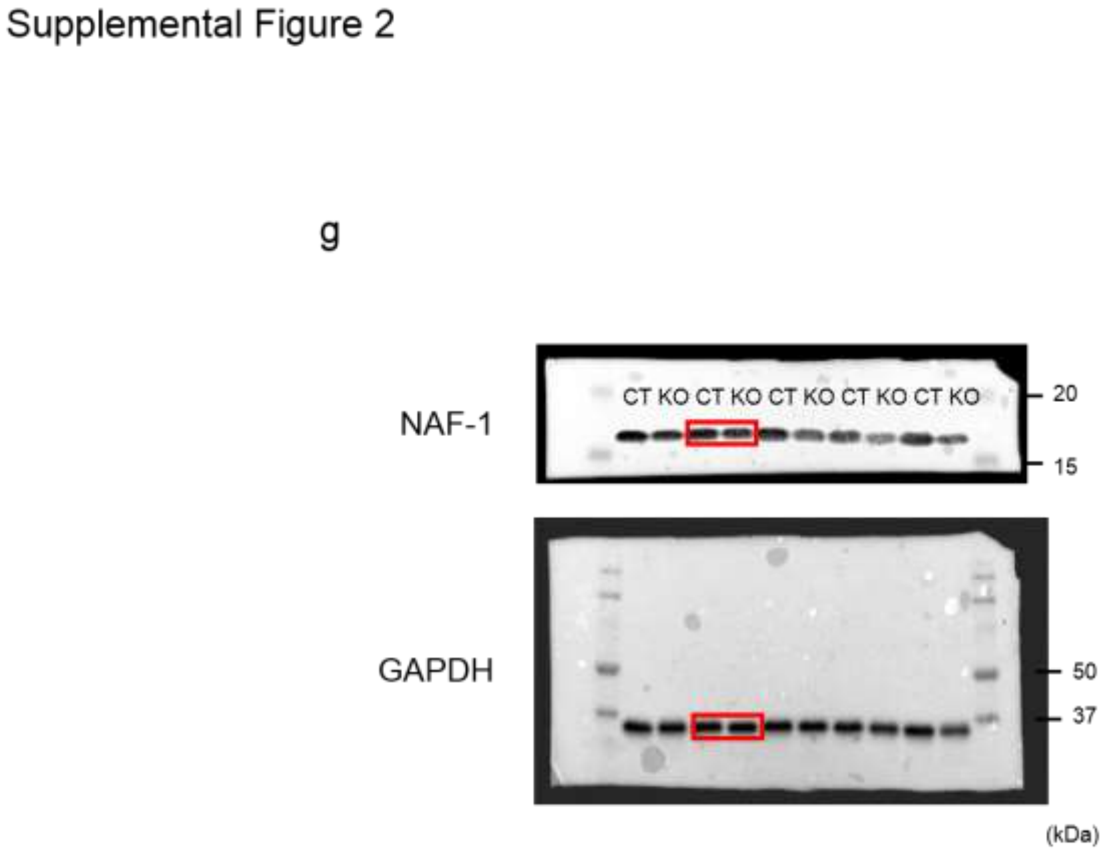

Original Western blots in Supplementary Figure 3a-d

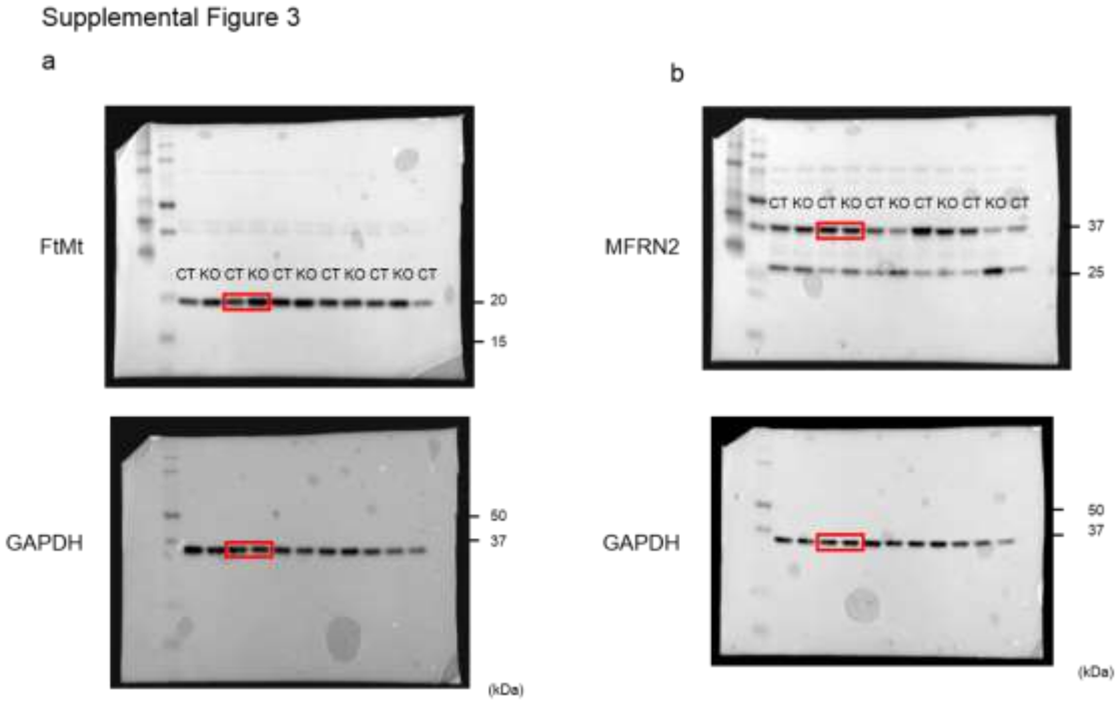

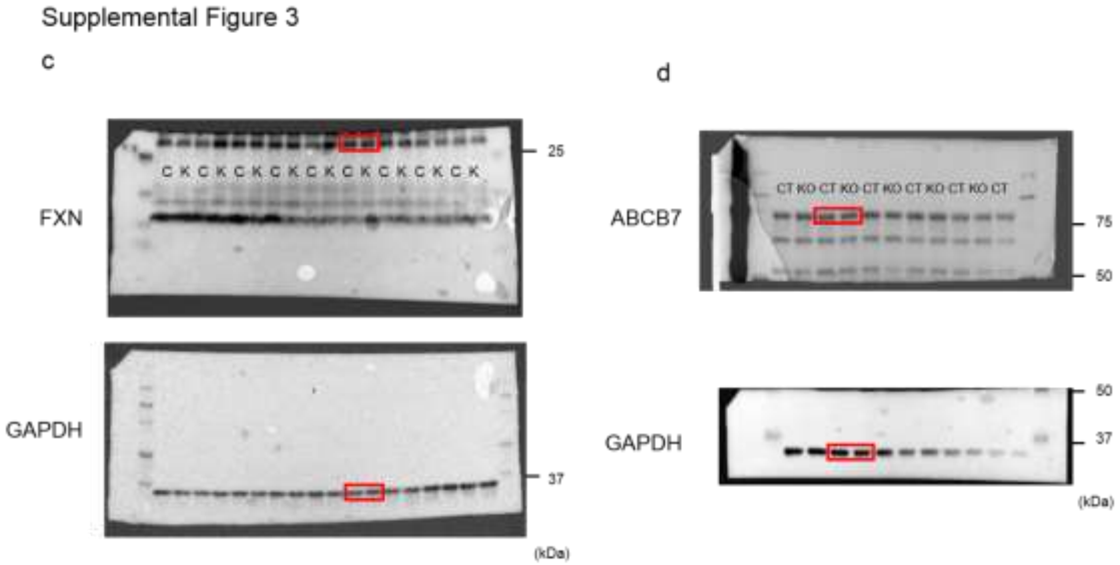

Original Western blots in Supplementary Figure 3e-h

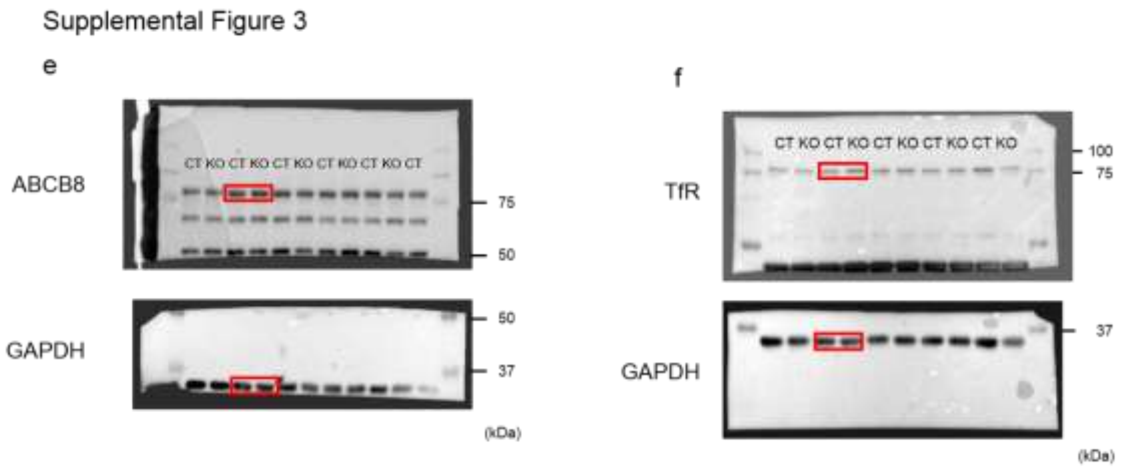

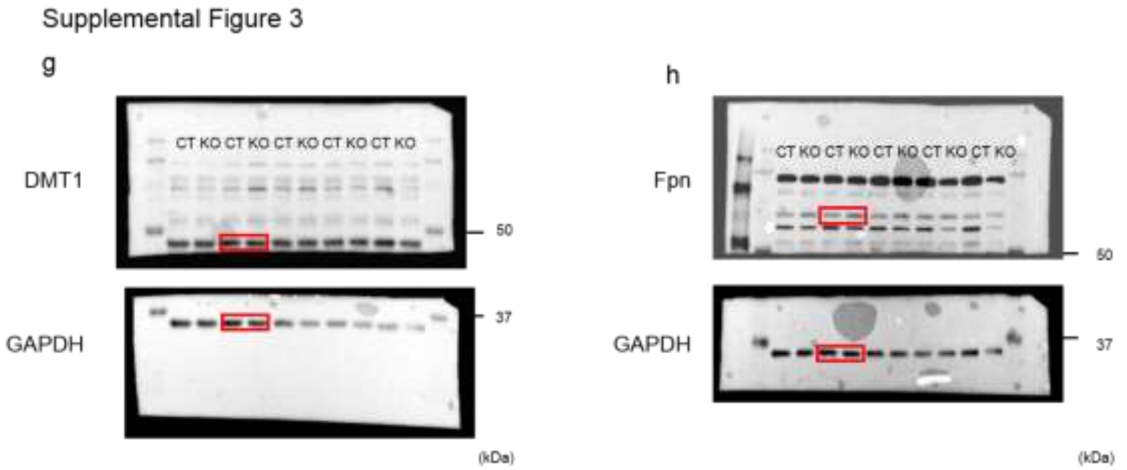

Original Western blots in Supplementary Figure 3i, j, m, n

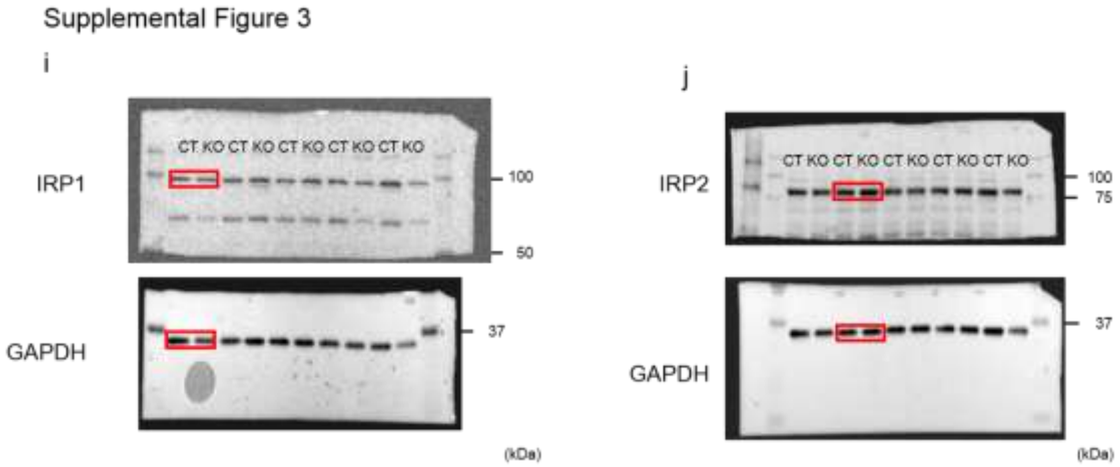

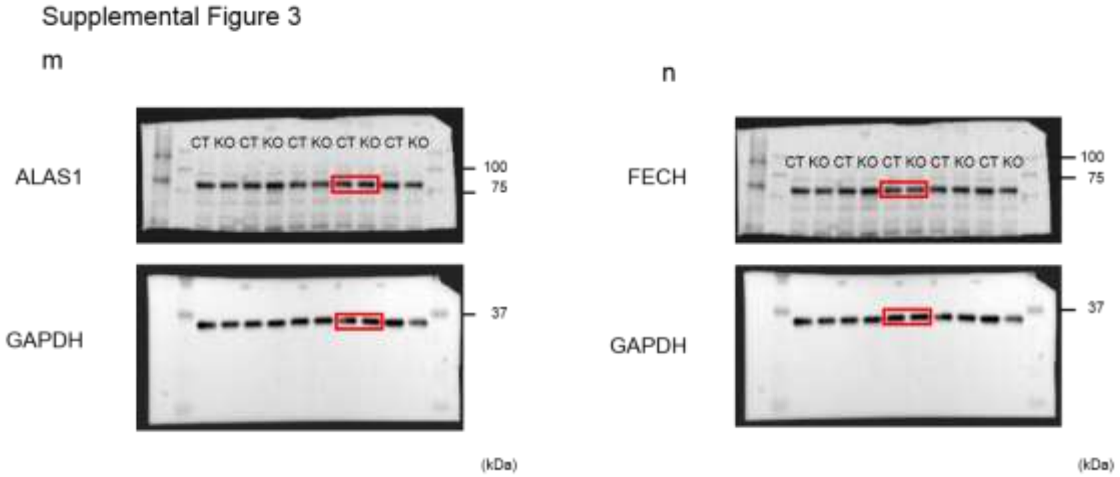

Supplement: Supplementary file 2 — Supplementary Information [file 42003_2021_1675_MOESM2_ESM.pdf]
