# Supplementary material for: Cardiac-specific loss of mitoNEET expression is linked with age-related heart failure
Source: Commun Biol. 2021 Jan 29;4:138. doi: 10.1038/s42003-021-01675-4 (PMC7846856; doi:10.1038/s42003-021-01675-4)
Supplement: Supplementary file 3 — Description of Additional Supplementary Files [file 42003_2021_1675_MOESM3_ESM.pdf]

Description of Additional Supplementary Files

File Name: Supplementary Data 1

Description: Raw data underlying plots in all figures including Figure 1-5 and Supplemental Figure 1-3 are available in Supplementary Data
