# Supplementary material for: Cardiac-specific loss of mitoNEET expression is linked with age-related heart failure
Source: Commun Biol. 2021 Jan 29;4:138. doi: 10.1038/s42003-021-01675-4 (PMC7846856; doi:10.1038/s42003-021-01675-4)
Supplement: Supplementary file 5 — Reporting Summary [file 42003_2021_1675_MOESM5_ESM.pdf]

nature research

Corresponding author(s): Shintaro Kinugawa

Last updated by author(s): Dec 4, 2020

Reporting Summary

Nature Research wishes to improve the reproducibility of the work that we publish. This form provides structure for consistency and transparency in reporting. For further information on Nature Research policies, see our Editorial Policies and the Editorial Policy Checklist.

Statistics

For all statistical analyses, confirm that the following items are present in the figure legend, table legend, main text, or Methods section.

n/a	Confirmed	
☐	☒	The exact sample size (*n*) for each experimental group/condition, given as a discrete number and unit of measurement
☐	☒	A statement on whether measurements were taken from distinct samples or whether the same sample was measured repeatedly
☐	☒	The statistical test(s) used AND whether they are one- or two-sided<br>*Only common tests should be described solely by name; describe more complex techniques in the Methods section.*
☒	☐	A description of all covariates tested
☐	☒	A description of any assumptions or corrections, such as tests of normality and adjustment for multiple comparisons
☐	☒	A full description of the statistical parameters including central tendency (e.g. means) or other basic estimates (e.g. regression coefficient) AND variation (e.g. standard deviation) or associated estimates of uncertainty (e.g. confidence intervals)
☐	☒	For null hypothesis testing, the test statistic (e.g. *F*, *t*, *r*) with confidence intervals, effect sizes, degrees of freedom and *P* value noted<br>*Give P values as exact values whenever suitable.*
☒	☐	For Bayesian analysis, information on the choice of priors and Markov chain Monte Carlo settings
☒	☐	For hierarchical and complex designs, identification of the appropriate level for tests and full reporting of outcomes
☒	☐	Estimates of effect sizes (e.g. Cohen's *d*, Pearson's *r*), indicating how they were calculated

Our web collection on statistics for biologists contains articles on many of the points above.

Software and code

Policy information about availability of computer code

Data collection	1. Western blot bands were analyzed using Bio-Rad ChemiDoc XRS+.<br>2. TaqMan quantitative real-time PCR was performed with a 7300 real-time PCR system (Applied Biosystems).<br>3. Mitochondrial respiratory capacity and hydrogen peroxide were measured with a high-resolution respirometer and spectrofluorometry (Oxygraph-2k-Fluorescence LED2-Module, Oroboros Instruments).<br>4. Mitochondrial iron content was measured with a commercial Iron Assay Kit (BioAssay Systems).<br>5. Mitchondrial electron micrographs were obtained using H-7100, Hitachi and JEM1400, JEOL.
Data analysis	All analyses were performed with GraphPad Prism 7 software.

For manuscripts utilizing custom algorithms or software that are central to the research but not yet described in published literature, software must be made available to editors and reviewers. We strongly encourage code deposition in a community repository (e.g. GitHub). See the Nature Research guidelines for submitting code & software for further information.

Data

Policy information about availability of data

All manuscripts must include a data availability statement. This statement should provide the following information, where applicable:
- Accession codes, unique identifiers, or web links for publicly available datasets
- A list of figures that have associated raw data
- A description of any restrictions on data availability

Raw data underlying plots in figures are available in Supplementary Data 1.

Field-specific reporting

Please select the one below that is the best fit for your research. If you are not sure, read the appropriate sections before making your selection.

☒ Life sciences ☐ Behavioural & social sciences ☐ Ecological, evolutionary & environmental sciences

For a reference copy of the document with all sections, see nature.com/documents/nr-reporting-summary-flat.pdf

Life sciences study design

All studies must disclose on these points even when the disclosure is negative.

Sample size	n=3-14 in each experiments
Data exclusions	Data were not excluded from the experiment unless apparent failures.
Replication	Each experiments were repeated more than 3 times to verify the reproducibility.
Randomization	In animal studies, we randomly allocated animals into groups.
Blinding	The entire experiment was shared by multiple researchers. Each experiment was conducted by researchers who did not know the group, and finally the data were integrated and analyzed.

Reporting for specific materials, systems and methods

We require information from authors about some types of materials, experimental systems and methods used in many studies. Here, indicate whether each material, system or method listed is relevant to your study. If you are not sure if a list item applies to your research, read the appropriate section before selecting a response.

Materials & experimental systems

n/a	Involved in the study	
☐	☒	Antibodies
☒	☐	Eukaryotic cell lines
☒	☐	Palaeontology and archaeology
☐	☒	Animals and other organisms
☒	☐	Human research participants
☒	☐	Clinical data
☒	☐	Dual use research of concern

Methods

n/a	Involved in the study	
☒	☐	ChIP-seq
☒	☐	Flow cytometry
☒	☐	MRI-based neuroimaging

Antibodies

Antibodies used	MITOL, ab77585, Abcam; Tom22, ab179826, Abcam; Tom40, sc-11414, Santa Cruz Biotechnology; Tom70, sc-390545, Santa Cruz Biotechnology; Miner1, #60758, CST; 4-hydroxynonenal, ab46545, Abcam; FtMt, ab111888, Abcam; MFRN2, ab80467, Abcam; FXN, ab175402, Abcam; ABCB7, ab151992, Abcam; ABCB8, ab133884, Abcam; TfR, ab84036, Abcam; DMT1, ab123085, Abcam; Fpn, ab85370, Abcam; IRP1, ab126595, Abcam; IRP2, ab80339, Abcam; 5'aminolevulinate synthase 1, ab84962, Abcam; ferrochelatase, ab55965, Abcam; GAPDH, #3683, CST
Validation	The validation of antibodies used for Western blot were performed by SDS-PAGE by the manufacturer, with relevant data presented on the manufacturer websites.

Animals and other organisms

Policy information about studies involving animals; ARRIVE guidelines recommended for reporting animal research

Laboratory animals	C57BL/6J mice, both sexes
Wild animals	C57BL/6J mice, both sexes
Field-collected samples	This study did not involve field-collected samples.
Ethics oversight	All experimental procedures and methods of animal care were approved by the Institutional Animal Care and Use Committee of National University Corporation Hokkaido University (Permit no. 16-0101) and conformed to the Guide for the Care and Use of Laboratory Animals published by the U.S. National Institutes of Health.

Note that full information on the approval of the study protocol must also be provided in the manuscript.
